# Account of Diseases in an Eastern District of London, from the 20th of September to the 20th of October

**Published:** 1800-11

**Authors:** 


*Account of Diseases in an Eastern District of London, from the 20th of September to the 20th of October.*

| | No. of Cafes. | | No. of Cafes. |
|---|---|---|---|
| ACUTE DISEASES. | | Chlorofis | 3 |
| Typhus | 15 | Fluor albus | 7 |
| Peripneumony | 5 | Amenorrhœa | 9 |
| Pleurify | 3 | Menorrhagia | 2 |
| Small-pox | 6 | Hemiplegia | 2 |
| Dyfentery | 10 | Cephalalgia | 5 |
| CHRONIC DISEASES. | | Vertigo | 4 |
| Cough | 15 | Chronic Eruptions | 15 |
| Cough with the Dyfpnœa | 12 | Chronic Rheumatifm | 10 |
| Hæmoptyfis | 6 | PUERPERAL DISEASES. | |
| Pleurodyne | 4 | Low Puerperal Fever | 3 |
| Phthifis Pulmonalis | 8 | Convulfions | 1 |
| Catarrh | 2 | Maftodynia | 4 |
| Diarrhœa | 22 | INFANTILE DISEASES. | |
| Enterodynia | 6 | Diarrhœa | 12 |
| Colica Pictonum | 1 | Herpes | 4 |
| Hypochondriafis | 5 | Aphthæ | 3 |
| Hyfteria | 4 | | |

Difeafes of the bowels, which have formed fo large a proportion of the lift in the late reports, ftill prevail very generally. Cholera, which a few weeks ago was fo very frequent in its attacks, and fo obftinate and alarming in its fymptoms, has not lately occurred; but Diarrhœas or Dyfenteries, in a flighter or more formidable manner, have vifited almoft every family. In many cafes where it has not conftituted the primary difeafe, *diarrhœa* has been an attendant fymptom; and fo general indeed has its appearance been, that it might be faid to blend and connect itfelf with almoft every difeafe. It ought not to be concealed, that in fome inftances it has proved highly falutary, and feems to have been the means which nature defigned to employ in order to effect a cure. It is the more proper to mention this, fince there is generally a ftrong defire on the part of the patient and his friends, too haftily to counteract this falutary procefs. In two or three cafes of incipient phthifis, during a copious inteftinal difcharge, the cough was much relieved, and the difficulty of breathing confiderably diminifhed.

Inftances of typhus are numerous. They are in general of the milder fpecies. The determination to the head is lefs violent, and the delirium lefs frequent than in former cafes.

By a ftatement taken from the Bills of Mortality, and fubjoined to this report, it appears that the Small Pox has proved uncommonly fatal. The unufual heat of the weather may be confidered as one caufe of this phenomenon. We will venture a conjecture, whether or not, in fome inftances, a long protracted hefitation, which of the two modes of inoculation to prefer, the varilous or the vaccine, has not expofed the party to the infection of the NATURAL Small Pox, and contributed to the great degree of fatality which has attended the difeafe.

The

The Deaths in the Bills of Mortality for the laſt three months are ſtated as follow:

| | | | |
|---|---|---|---|
| Abſceſs | 8 | Jaundice | 17 |
| Abortive | 10 | Inflammation | 117 |
| Aged | 281 | Lunatic | 24 |
| Apoplexy | 15 | Meaſles | 46 |
| Aſthma | 56 | Mortification | 58 |
| Cancer | 10 | Palſy | 31 |
| Child Bed | 35 | Pleuriſy | 8 |
| Conſumption | 1060 | Small Pox | 848 |
| Convulſions | 951 | Still Born | 90 |
| Croup | 4 | Suddenly | 23 |
| Dropſy | 163 | Teething | 72 |
| Fever | 675 | Thruſh | 5 |
| French Pox | 4 | Water in the Head | 15 |
| Gout | 28 | Worms | 5 |
| Hooping Cough | 44 | | |

